# Factors Affecting Gluten-Free Dietary Adherence in Patients with Neurological Gluten-Related Disease

**DOI:** 10.3390/nu18030480

**Published:** 2026-02-01

**Authors:** Iain D. Croall, Marios Hadjivassiliou, David S. Sanders, Nick Trott, Nigel Hoggard

**Affiliations:** 1 School of Medicine and Population Health, University of Sheffield, Sheffield S10 2RX, UK; 2 Department of Neurology, Sheffield Teaching Hospitals NHS Foundation Trust, Sheffield S10 2JF, UK; 3 Academic Unit of Gastroenterology, Sheffield Teaching Hospitals NHS Foundation Trust, Sheffield S5 7AT, UK; david.sanders1@nhs.net (D.S.S.);

**Keywords:** coeliac disease, gluten ataxia, gluten-free diet, gluten neuropathy, gluten encephalopathy, neuro coeliac disease, neurological gluten-related disorders

## Abstract

**Background/Objectives**: The gluten-free diet (GFD) is the primary treatment for patients with neurological gluten-related disease, which may occur with or without coeliac disease (CD). Dietary adherence is arguably most important in such patients, as ongoing gluten exposures have been shown to exacerbate irreversible neurological deterioration. We utilised a cross-sectional postal questionnaire to explore factors affecting dietary adherence in a large sample of such patients, highlighting potential areas of dietetic need. **Methods**: Patients returned a postal questionnaire (N = 225), which assessed self-reported GFD adherence by the Biagi scale and a visual analogue scale. CD status was ascertained, alongside symptomatology and mood (via the Hospital Anxiety and Depression Scale). Dietary knowledge was tested by a “quiz” where respondents identified which of 10 foodstuffs should be avoided on a GFD. **Results**: Self-reported adherence was high across the cohort, but was significantly higher in those with CD than those without. Patients with CD more often reported a number of gastrointestinal symptoms as acute reactions if they were to eat gluten. Similarly, the CD subgroup reported greater overall acute discomfort following gluten, while across the cohort greater such discomfort correlated with greater dietary adherence. Overall, 6.2% of the participants both reported strict diets (scoring ≥ 90 on the visual analogue scale) but via the quiz indicated an erroneous belief that they could eat a gluten-containing foodstuff. Lower adherence was correlated with higher depressive scores, with post hoc analyses finding that this was driven by patients without CD. **Conclusions**: This study highlights a need for increased dietary support in patients with neurological gluten sensitivity, particularly when there is no co-diagnosis of CD. Therapies targeting depression may additionally bolster dietary adherence.

## 1. Introduction

Gluten sensitivity exists as a spectrum of autoimmune conditions that can target multiple organ systems [1]. Coeliac disease (CD) is the best understood condition under this umbrella and its associated enteropathy is well-characterised [2]. Less researched are neurological forms of gluten sensitivity, Neurological Gluten-Related Disorders (NGRDs), which primarily include phenotypes of ataxia, neuropathy and encephalopathy [3,4]. In gluten ataxia the cerebellum undergoes marked loss of Purkinje cells, resulting in atrophy and predominantly causing disorders of balance and motor coordination [5]. Patients with gluten neuropathy experience peripheral sensory disturbances [6], while cases of gluten encephalopathy are defined by severe headache and cognitive dysfunction [3]. These neurological manifestations may occur alongside CD or in isolation without enteropathy. Regardless, the primary treatment is the same as for CD; strict adherence to the gluten-free diet (GFD), which has been shown to benefit patients via objective brain MRI outcomes [7,8].

The pathogenesis of NGRDs is not fully understood, although evidence increasingly implicates infiltration of certain gluten-related antibodies into the central nervous system, with possible downstream inflammatory damage [9]. Accordingly, a diagnosis depends on the detection of an autoimmune response to gluten. This is similar to CD, where antibodies to transglutaminase 2 (TG2, commonly called TTG) are made and it is the presence of these TG2 antibodies that forms a critical part of the diagnostic process for CD. For NGRDs, TG2 antibodies are still checked, but antibodies against gliadin [10], and more recently against transglutaminase 6 (TG6), are additionally assayed. These biomarkers (particularly IgA TG6 antibodies) have been shown to be highly over-represented in patients with “sporadic” ataxia/neuropathy [11,12,13], and their presence and persistence is associated with increased disease severity in patients with neurological gluten-related disorders regardless of a co-diagnosis of CD [14].

In patients with classic CD, gluten exposures are undesirable but not necessarily permanently detrimental. The gut has good healing ability and, once gluten is eliminated, typically recovers very effectively. By comparison and in all practical terms, the brain does not have the ability to heal. Patients may enjoy a modest restoration of some function if underlying disease processes cease, but this is largely through the dissipation of the acute disease state followed by adaptive processes; brain tissue does not in any meaningful way regenerate, and fundamentally severe injury will remain fundamentally severe. Strict adherence to the GFD is therefore of the upmost importance in these patients to prevent avoidable neurological deterioration and the associated accumulated disability.

The above research underlines that achieving negativity of circulating antibodies should be the goal of treatment. Research into what magnitude and frequency of gluten ingestion would maintain antibody positivity is scarce and challenging by nature, given the likely variability in serological responses between individuals and also between gluten-related antibodies. One study found that high compliance with the GFD led to negative gliadin tests after 6 months, but only identified low compliance as when a participant reported knowingly eating gluten during the study [15]. Another report has confirmed that the GFD reduces TG6 titre/positivity in a sample of patients with dermatitis herpetiformis [16], although degree of dietary adherence was not measured. Patients with neurological gluten-related disorders who self-report strict diets are more likely to become negative for IgA TG6 on follow-up serology, but over half of this apparently compliant group remain positive [14]. One paper found no change in TG6 positivity between treated and untreated patients with CD, in contrast with TG2, which, as would be expected, appeared largely eliminated by dietary adherence [17]. It seems that a particularly strict GFD beyond what would be considered adequate for “typical” CD might be required to resolve the neurological autoimmune state, and it would therefore be advisable that patients should adhere in the strongest terms until confirmed otherwise. This also highlights the utility of routine serological testing in the monitoring of patients with neurological gluten-related disease, as the most direct way of assessing if a person’s immune reactivity has resolved or not.

Although some questions of serological response have been investigated, dietary attitudes have not been studied in patients with NGRDs. The rate of “strict” GFDs, with respect to conventional benchmarks, is not known, nor are what patient factors affect this.

While tools such as the Biagi scale [18] inevitably suffer from some error introduced by self-reporting variability, these types of inventories also give direct insight into a patient’s understanding of their treatment and disease burden, and are therefore highly relevant for informing services such as dietetics. In CD, numerous reports have linked poorer adherence to depressive symptoms, though it is difficult to know which of these is causative of the other [19]. It is possible that symptomatology may influence adherence. While a diagnosis of an NGRD consistently relies on the same symptomatic criteria, it might therefore be expected that the greatest inter-patient difference in this group is driven by whether the patient also suffers from CD. This would separate those who differently experience gastrointestinal discomfort, which in turn may change the levels of motivation patients feel to avoid acute symptomatic flares.

In order to understand these details we performed a postal questionnaire assessing dietary compliance, attitudes, mood and symptomatology in a cohort of patients with diagnosed NGRDs (with and without CD). Our results should provide important context for understanding treatment attitudes in this group, and help better refine dietetic input in this unique clinical scenario where a dietary intervention ameliorates neurological dysfunction.

## 2. Materials and Methods

### 2.1. Participants

The study was based at a specialist neurological clinic in Sheffield, UK, that diagnoses and cares for patients with NGRDs, with and without CD. The diagnostic criteria for patients are:

- Neurological symptoms in keeping with gluten-related phenotypes (i.e., ataxia, neuropathy or encephalopathy, or some combination of these);
- Confirmation of relevant neurological disease via appropriate clinical testing (brain MRI, nerve conduction studies, etc.);
- Positivity for gluten related antibodies (IgA/IgG gliadin or TG6, or IgA TG2 or endomysial antibodies);
- All other causes excluded following exhaustive investigations, which include as examples CSF analysis, genetic panels and testing for neuronal antibodies such as anti-GAD65, anti-Hu and anti-Yo.

In this way, all participants at the time of their invitation to take part in the study held a diagnosis of a NGRD. Some patients had a co-diagnosis of CD. This may be an initial diagnosis via gastroenterology, with the subsequent development of neurological symptoms warranting a referral to this clinic. Alternatively, neurological problems may have been the primary presentation and subsequent investigations identified CD. Patients without CD show evidence of gluten sensitivity by seropositivity for either/or gliadin and TG6 antibodies. Upon diagnosis, all patients were advised to go on a strict gluten-free diet and were provided with dietetic support.

### 2.2. Data Collection

For the duration of the study (between May of 2022 and September of 2023), postal questionnaires were consecutively sent to all patients who had an upcoming routine clinical appointment on a rolling weekly basis. The Questionnaire S1 (included in the Supplementary Materials) combined a number of both validated and bespoke components that were relevant to the current study and assessed the following:

- Self-reported demographic information, including if the patient also had CD and the length of time since the patient was diagnosed with their initial gluten-related disorder.
- Dietary adherence: This was measured in two ways. The first was via the Biagi scale (with the final question adapted to reflect UK organisations by asking if the patient only eats packaged food guaranteed by Coeliac UK). Participants were classified as Biagi “adherent” if they scored ≥3. The second was via a visual analogue scale (VAS), which asked the patient to indicate with a mark on a 10 cm line how strict their diet was, between “I make no effort to restrict gluten” and “I am as strict as anyone could be”; the position of the mark was measured in mm from left to right, and therefore converted to a number between 0 and 100, with 100 representing maximum adherence.
- Dietary knowledge: A “quiz” component of the questionnaire presented participants with ten foodstuffs and asked them to select all of those which should be avoided on a gluten-free diet; the three correct answers were wheat, rye and barley; respondents who failed to identify at least one of these three options would be indicated as being at risk of unknowingly eating gluten.
- Acute symptomatology: Patients were asked via tick boxes to indicate which of a selection of symptoms they would expect to experience if they ate gluten. The selection was adapted from the Celiac Symptom Index [20] but adapted to include additional neurological symptoms of interest, therefore including extra-intestinal (headaches, balance problems, movement problems, sensory disturbances, restless legs, brain fog, fatigue, skin rash, irritability), and gastrointestinal phenomena (abdominal pain, diarrhoea, bloating, constipation, vomiting, mouth ulcers). Participants additionally indicated by a single VAS the expected overall severity of their symptoms during an acute gluten reaction, with the line ranging between “barely noticeable” to “as bad as they could be” (designed and quantified in the same manner as the other already described).
- Mood: The Hospital Anxiety and Depression Questionnaire [21] provided scores reflecting degree of anxiety and depressive symptoms, with cut-offs available to indicate suspicion of overt clinical dysfunction.

The questionnaire included other components which were not analysed in the current paper. The project sought to recruit the maximum possible number of patients for the duration of the study. Due to the novelty of the data, the analyses were considered exploratory.

### 2.3. Statistical Analysis

All statistics were performed in SPSS version 29.

Key variables were summarised to determine frequency of CD, rate of depression/anxiety, dietary knowledge and dietary adherence. Statistical analyses compared dietary adherence, symptomatology and mood between those with and without CD. Adherence was further correlated with raw scores for depression/anxiety and expected symptom severity of acute gluten reactions. All variables were inspected for normality to inform as to whether parametric or non-parametric testing should be used. Groupwise analyses accordingly used either independent *t*-tests or Mann–Whitney U, while correlations used either Pearson or Spearman. Analyses involving binary groupings and binary outcomes were performed by X^2^.

Participants with partially completed data were excluded on a per-analysis basis as necessary. For example, the HADS scores and outcomes require all contributing questions to have been completed.

## 3. Results

A total of 576 questionnaires were posted, with 225 returned. The sample size of completed returned data on a per-outcome basis was as follows: presence of different symptoms N = 221, dietary quiz N = 223, HADS depression N = 220, HADS anxiety N = 220, Biagi scale N = 219, VAS dietary adherence N = 222, VAS acute symptom severity N = 157.

Participants were, on average, 61.2 ± 14.1 years old and 63.6% female. Gluten ataxia was the most common primary diagnosis affecting 65.8% of respondents, with gluten encephalopathy accounting for 18.9% and gluten neuropathy for 15.3%. The rate of self-reported CD was 40.6%, which broadly matches other published data from this clinic, which found positive coeliac antibody tests (TTG or EMA) to be present in 32.7% of patients via monitoring over a 5+ year interval [14]. Age and sex were not significantly different between respondents based on the presence of CD. Patients with CD reported a significantly longer duration of time since their original gluten-related diagnosis (8.8 ± 7.7 years vs. 4.2 ± 4.5 years, *p* < 0.001). The presence of (any) other self-reported neurological condition, or a score on the HADS indicating clinical depression, were not significantly different between CD/non-CD subgroups. This information is summarised in Table 1 below.

### 3.1. Dietary Adherence and Education

**Figure 1:**
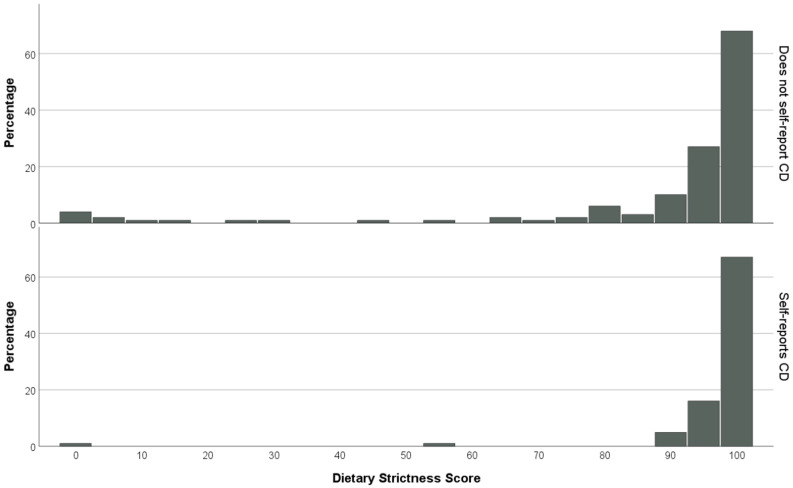
Histograms demonstrating responses to a visual analogue scale which assessed self-reported dietary strictness, separated by coeliac disease diagnosis. A higher number indicates greater adherence. Participants with coeliac disease reported significantly greater adherence values.

According to the Biagi scale, 82.6% of patients were adherent to the GFD; 8.2% obtained a score of 0, indicating that they ate quantities of gluten voluntarily and often. Biagi adherence was not significantly different between participants with and without CD (*p* = 0.101). The VAS dietary strictness score showed a heavy skew towards strict diets, with a median score of 98. Using a score of 90 on the VAS as an exploratory cut-off for “strict” diets, 14.4% gave a score under this and 1.4% of participants gave a score of 0. Mann–Whitney U analysis did show a significant difference in VAS dietary adherence, whereby participants with CD gave higher scores (median = 10) than those without CD (median = 9.8, *p* < 0.001). This is visualised in Figure 1.

The dietary quiz found that 39.5% of participants gave perfect responses, i.e., identified only wheat, rye and barley as being the foodstuffs that should be avoided. The majority of remaining participants were overcautious and included additional other options, thereby still indicating full gluten avoidance. The rate of participants who failed to identify at least one of wheat, rye or barley was 9.9%. This was not significantly different between those with and without CD, but was “approaching” significance (X^2^ *p* = 0.066), with 5.5% of the CD group failing vs. 13.0% of the non-CD group. To account for patients who may not follow the diet regardless, across the whole cohort, 6.3% of participants indicated reasonable adherence (VAS score ≥ 90) but failed this quiz in a manner which suggests they eat gluten.

### 3.2. Symptomatology and Diet

The following lists the symptoms reported as an acute reaction to gluten by order of prevalence, as given across the whole study cohort: bloating (34.7%), diarrhoea (34.2%), abdominal pain (33.8%), balance problems (29.7%), brain fog (28.6%), fatigue (27.9%), headaches (20.7%), movement problems (18.9%), sensory disturbances (16.7%), irritability (16.2%), vomiting (14.4%), restless legs (11.7%), mouth ulcers (11.7%), skin rash (9.5%), constipation (8.6%).

Table 2 shows the frequency of these according to whether or not the participant reported a diagnosis of CD, and the results of X^2^ comparisons between these groups. To summarise, a number of abdominal symptoms were reported at higher rates in the CD group (abdominal pain, diarrhoea and vomiting all at *p* < 0.001, and bloating and mouth ulcers at *p* < 0.05). Irritability was also more prevalent in the CD subgroup (*p* = 0.041). The findings for abdominal pain, diarrhoea and vomiting would survive Bonferroni correction if applied.

**Figure 2:**
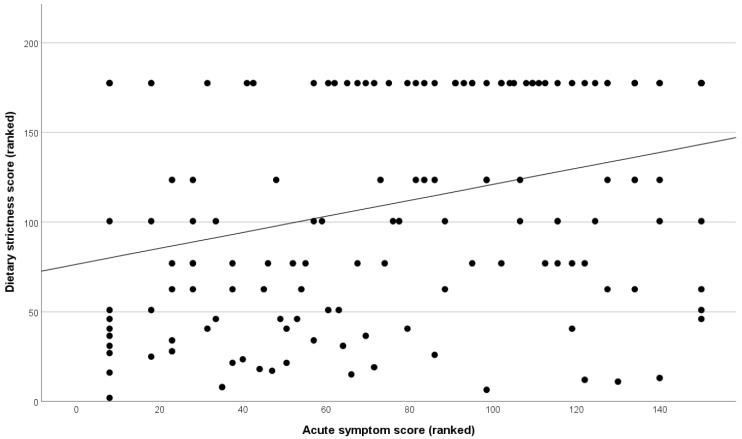
Scatterplot visualising the significant positive correlation between acute symptom severity and dietary strictness in patients without CD. Data has been transformed to rank cases for visualisation only, reflecting the Spearman correlation analysis.

Across the whole group, VAS scores concerning the severity of expected symptoms as an acute reaction to gluten were not significantly different when compared between participants who were and were not adherent to the diet according to Biagi response (*p* = 0.125). They did, however, show a highly significant positive (Spearman) correlation with VAS dietary adherence (r = 0.331, *p* < 0.001, Figure 2), with worse expected reactions predicting greater adherence. The acute symptom severity score was also significantly different based on CD diagnosis, wherein those with CD reported worse severity (median = 8.5) than those without (median = 5.6, Mann–Whitney U, *p* < 0.001).

### 3.3. Mood and Diet

According to the HADS, the rate of participants who met the criteria for clinical anxiety was 31.8%. The rate of participants who met the criteria for clinical depression was 20.0%. Raw scores for anxiety and depression were not significantly different between patients with and without CD (independent samples *t*-test; anxiety *p* = 0.889, depression *p* = 0.327).

**Figure 3:**
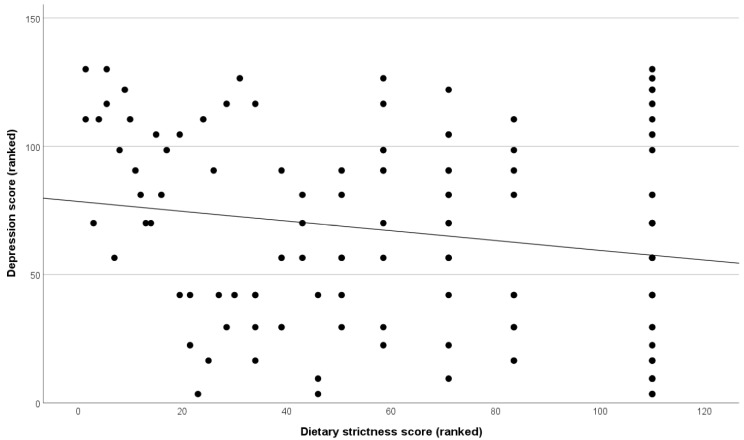
Scatterplot visualising the significant negative correlation between depression and dietary strictness in patients without CD. Data has been transformed to rank cases for visualisation only, reflecting the Spearman correlation analysis.

Depression scores were significantly lower in patients who were adherent according to the Biagi scale (6.7 ± 4.1) than those who were not adherent (8.3 ± 4.1, *p* = 0.036). This effectively uses a larger sample size to replicate a previously published analysis on a smaller subgroup of this cohort (where at least 5 years of accompanying serological data are additionally available) [14]. Biagi adherence did not predict anxiety scores. In the current dataset, VAS dietary adherence showed a significant negative correlation with the depression score, wherein greater dietary strictness was related to lower depressive symptoms (r = −0.161, *p* = 0.018). Further analyses found this was not present in the CD subgroup but was driven by those without CD, where the association remained significant (r = −0.184, *p* = 0.036, Figure 3). Anxiety did not correlate with VAS dietary adherence.

### 3.4. Multivariate Analysis

A multivariate (linear) model was constructed using factors indicated in univariate analyses to be relevant to dietary adherence. The outcome was dietary strictness (as measured by the VAS), with age, sex, CD status, HADS depression score and acute symptom severity score as predictor variables. Both VAS variables were ranked before being entered into the model to account for their non-normal distribution. The result showed the acute symptom severity variable to survive the model with *p* = 0.001. CD status and age both approached significance at *p* = 0.055 and *p* = 0.077, respectively.

## 4. Discussion

NGRDs involve brain injury that occurs as a result of gluten ingestion [3]. This creates a relatively unique clinical scenario in which potentially devastating neurological problems may be treated with a dietary intervention. The GFD is recommended to such patients whether they have a co-diagnosis of CD or not, but despite the importance of the intervention, it is not known how strictly these patients follow it or what factors affect their adherence. In this study, which recruited neurology patients who receive regular clinical review and dietician support, we find mostly high self-reported dietary strictness, but we also find that intertwined factors concerning the coexistence of CD, the expected severity of symptomatic response to gluten ingestion, and depressive symptoms may impact this. We also report a modest rate of dietary misconception. Overall, these results highlight a need for further research in this area and potentially for dietetic guidance to be refined in these patients so that treatment effectiveness can be bolstered and patients more protected against irreversible neurological deterioration.

The mechanism of injury in conditions such as gluten ataxia is not fully understood but is hypothesised to be due to an autoimmune reaction following gluten ingestion [3], which involves the production of gluten-related antibodies [9]. While this is somewhat analogous to CD, mounting research indicates that the specific antibodies produced in these reactions differ, thus explaining why CD and NGRDs may present together or in isolation of one another. Antibodies to TG6 have been indicated as a marker of patients at risk of gluten-related neurological disease [12], and we recently published data from this cohort showing that history of IgA TG6 is associated with increased disease severity [14].

Following the GFD is the primary treatment, and research has linked achieving antibody negativity to improved clinical outcomes. We have previously shown that patients who become negative for gluten antibodies have a slower rate of cerebellar atrophy [7] and enjoy a recovery of brain biomarkers which reflect healthy neuronal functioning [8]. As the brain does not recover from atrophy, preventing progression of these diseases wherever possible is of the upmost importance. It is essential that areas of dietary need are identified and addressed.

While previous research has demonstrated that the diet is as necessary for those without CD, such patients will nonetheless have a different lived experience of their condition. Neurological injury such as the cerebellar atrophy of gluten ataxia does not necessarily occur alongside acute symptomatic flares. In this way, patients who have a co-diagnosis of CD may have additional intrinsic motivation to avoid gluten given the immediate abdominal discomfort they would typically experience if they ate it. Our findings reflect this. While adherence to the diet was generally high across the cohort, self-reported dietary strictness was significantly greater in patients with CD than those without. Further, various gastrointestinal symptoms (as acute gluten reactions) were reported at substantially higher rates in patients with CD, with the overall severity of their acute symptoms also being greater. This severity was significantly correlated with self-reported dietary adherence, and was the only variable to remain significant in a multivariate model predicting strictness, indicating it to be the most relevant single factor. By comparison, while some neurological phenomena like brain fog, headaches and balance problems were also reported at moderately high rates these did not differ based on CD status. Our findings therefore show that patients with greater symptomatic burden after eating gluten are more likely to adhere strictly to the diet, with this accordingly meaning that patients without CD, who generally lack gastrointestinal symptoms and experience overall milder acute flares, may adhere less. These patients might therefore be the focus of particularly targeted dietary education.

The HADS questionnaire shows evidence of mood disorder at high rates in these patients. Although we do not have a control group, comparable UK data suggests that the threshold for anxiety was reached approximately twice as often as in normative samples, while depression was reached approximately thrice as often [22], likely demonstrating a considerable quality of life impact from this disease. Reflecting the results in some of the previous literature [19], we also found a significant relationship between level of depression and dietary adherence, an association notably driven in our data by patients without CD. Untangling the cause/effect of such findings is not feasible without further study, but it is important to understand whether heightened depression may in part be driven by more active disease due to lower dietary adherence. This is highlighted as an area for further study, while our data otherwise suggests that greater psychological attention may be warranted in these patients.

In an exploratory component our survey also assessed dietary knowledge via a multiple-choice “quiz”. A tenth of the group failed to identify wheat, rye or barley as being a foodstuff that should be avoided on a GFD; approximately 6% of the overall group failed this quiz in a manner which suggests they erroneously eat gluten yet self-reported relatively high levels of dietary adherence. The need for greater dietary education is therefore highlighted to ensure that those who are attempting a strict diet are equipped and able to do so. While not as immediately concerning, the majority of quiz responses showed an overcautiousness around foodstuffs that should be avoided. Such hypervigilance would also ideally be addressed through dietician input, as patients may be on unnecessarily restrictive diets and would enjoy more food choice.

Dietary adherence was investigated by both the Biagi scale and a bespoke VAS, which provided continuous data rather than categorical outcomes. GFD strictness is difficult to assess and self-reported measures are known to often have poor experimental sensitivity with respect to objective disease markers (such as villous atrophy in patients with CD [23]). Notably, in our study it was the VAS strictness score that achieved statistical significance in a number of analyses where the Biagi score did not, possibly indicating greater statistical sensitivity despite remaining a quick and simple self-reported assessment. This was the case for finding overall differences in adherence between respondents with vs. without CD, and also for finding an association between dietary strictness and the severity of acute reactions to gluten. Finally, patients with CD reported a longer disease duration (with respect to their initial gluten-related diagnosis), reflecting the tendency for adult CD to be diagnosed earlier than an adult NGRD that occurs without CD.

The study has limitations. Self-reported data has a number of drawbacks, such as differing interpretation of survey questions from one responder to another, which ultimately affects the accuracy of information collected. Self-reporting CD is not entirely reliable, although alternatives such as biopsy-confirmations from medical notes suffer different issues, such as if a patient was following an adequate gluten challenge at the time of the procedure. As referenced, data from a larger (overlapping) patient cohort from this clinic [14] indicates CD to be present at a similar rate to what was self-reported here; longitudinal contemporaneous serology is only available in a subset of the questionnaire respondents and so was not analysed here. Self-reporting information, while undesirable, would generally be expected to increase data noise, making significant findings harder to achieve. While there is therefore greater possibility of false negative findings, confidence in the results given should remain strong. The study did not have formal power calculations, but the presence of multiple statistical findings similarly indicates sufficient power to detect many effects. It is possible that the dietary quiz may overestimate the degree of people inadequately following the diet if some responders simply did not attend to the options as closely as needed on the day. Proper engagement with a dietician is needed to ascertain instances of genuine dietary misunderstanding. Some aspects of the questionnaire were bespoke, which, while necessary to study the unique clinical context, raises questions of validation. Bespoke items were designed using conventional approaches (notably via VAS) to ensure maximum data sensitivity. Results were not subject to multiple comparisons correction (although, as highlighted, a number of findings at high significance thresholds would survive this); formal correction was not performed due to the exploratory nature of the data combined with the unique clinical context. Future studies should build on this initial work to focus on more specific associations indicated here and further confirm associations. Finally, as this data is from a single specialised clinic, information may not be representative of other centres.

## 5. Conclusions

In conclusion, our study highlights that patients who have NGRDs without a co-diagnosis of CD may require greater dietetic focus. A relative lack of acute symptomatic response appears to lower dietary adherence in these patients, while a relationship between this poorer adherence and depressive symptoms is also established. Our data cannot untangle which of these is causative of the other, but given the high rates of apparent depression and anxiety in this cohort, psychological intervention may be warranted. Finally, we show that a modest subgroup of patients might be failing to follow the diet despite believing that they are, further demonstrating the need for close dietetic involvement. Neurological conditions can seldom be addressed by accessible dietary changes, and these opportunities should not be missed once patients who stand to benefit have been diagnosed. This observational study provides important groundwork in determining whether changes to dietetic input are required for patients with NGRDs.

## Figures and Tables

**Table 1:** Descriptive statistics of respondents summarised by the presence of CD. Results of statistical testing (either independent *t*-tests or X^2^ analysis) is also included, indicating patients with CD to have a significantly longer period of time since their initial diagnosis of a gluten-related disorder.

Variable	Patients with CD	Patients Without CD	Test Statistic (t Value or X^2^ Value)	*p* Value
Age	60.4 ± 13.8	61.6 ± 14.2	0.590	0.556
Sex (% female)	68.2%	60.2%	1.483	0.223
Time since original gluten-related diagnosis (years)	8.8 ± 7.7	4.2 ± 4.5	5.548	<0.001
Presence of other neurological diagnoses (% yes)	47.3%	41.4%	0.764	0.382
Indicated to have depression (i.e., HADS-D score > 10)	14.8%	22.9%	2.204	0.138

**Table 2:** Rate of symptoms expected as an acute reaction to gluten in patient subgroups based on the existence of a CD diagnosis. Data are compared between groups by X^2^ analysis with accompanying statistics (*p* values are uncorrected for multiplicity). Significant results are indicated by *.

Symptom	Prevalence in Patients with CD	Prevalence in Patients Without CD	X^2^ Value, *p* Value
Headaches	25.8%	17.4%	2.286, 0.131
Balance Problems	29.2%	30.3%	0.030, 0.862
Movement Problems	18.0%	19.7%	0.102, 0.749
Sensory Disturbances	16.9%	16.7%	0.001, 0.971
Brain Fog	32.1%	26.2%	0.792, 0.374
Restless Legs	11.2%	12.1%	0.040, 0.841
Fatigue	34.8%	23.5%	3.391, 0.066
Abdominal Pain	48.3%	24.2%	13.740, <0.001 *
Diarrhoea	49.4%	24.2%	14.957, <0.001 *
Bloating	44.9%	28.0%	6.698, 0.010 *
Irritability	22.5%	12.1%	4.177, 0.041 *
Constipation	10.1%	7.6%	0.435, 0.509
Vomiting	24.7%	7.6%	12.616, <0.001 *
Mouth Ulcers	19.1%	6.8%	7.726, 0.005 *
Skin Rash	13.5%	6.8%	2.746, 0.097

## Data Availability

Data is not publicly available as the ethics of the study do not permit this. Anonymised data may be made available on reasonable request to the corresponding author and subject to the relevant approvals.

## Supplementary Materials

The following supporting information can be downloaded at https://www.mdpi.com/article/10.3390/nu18030480/s1, Study Questionnaire S1.

## Abbreviations

The following abbreviations are used in this manuscript:

GFD	Gluten-free diet
CD	Coeliac disease
NGRD	Neurological gluten-related disorders
TG2	Transglutaminase 2
TTG	Tissue transglutaminase
TG6	Transglutaminase 6
IgA	Immunoglobulin A
HADS	Hospital anxiety and depression scale
VAS	Visual analogue scale
